# Dr. T. B. McClintic

**Published:** 1912-09

**Authors:** 


Dr. T. B. McClintic of the U. S. Marine Hospital and Public Health Service, died Aug. 13, at Washington, D. C., aged 39. He was born in Warm Springs, Va., graduated in medicine at the University of Virginia and appointed to the service, 15 years ago. For the last two years, he had been studying the Rocky Mountain Spotted Fever, first well known through the researches of Chowning and Wilson, who traced its transmission to a squirrel tick. Dr. McClintic had labored especially in the Bitter Root valley and so successfully that no case of the fever had occurred this year until he himself developed symptoms on Aug. 9, and started for Washington, typic signs appearing en route. Mere words are inadequate praise for a martyr. In the past, there has been a tendency on the part of a few narrow minded army and naval officers to sneer at the Marine officers as civilians but this feeling has almost entirely disappeared and, indeed, even in the army and the navy, the risks especially of medical officers, are constant and not dependent on the presence of war.

